# Hydrothermal synthesis of nanostructured graphene/polyaniline composites as high-capacitance electrode materials for supercapacitors

**DOI:** 10.1038/srep44562

**Published:** 2017-03-14

**Authors:** Ronghua Wang, Meng Han, Qiannan Zhao, Zonglin Ren, Xiaolong Guo, Chaohe Xu, Ning Hu, Li Lu

**Affiliations:** 1 College of Materials Science and Engineering, Chongqing University, No. 174 Shazhengjie Road, Chongqing 400044, P.R. China; 2 College of Aerospace Engineering, and The State Key Laboratory of Mechanical Transmissions, Chongqing University, No. 174 Shazhengjie Road, Chongqing 400044, P.R. China; 3 Department of Mechanical Engineering, National University of Singapore, 2 Engineering Drive 3, Singapore 117581, Singapore

## Abstract

As known to all, hydrothermal synthesis is a powerful technique for preparing inorganic and organic materials or composites with different architectures. In this reports, by controlling hydrothermal conditions, nanostructured polyaniline (PANi) in different morphologies were composited with graphene sheets (GNS) and used as electrode materials of supercapacitors. Specifically, ultrathin PANi layers with total thickness of 10–20 nm are uniformly composited with GNS by a two-step hydrothermal-assistant chemical oxidation polymerization process; while PANi nanofibers with diameter of 50~100 nm are obtained by a one-step direct hydrothermal process. Benefitting from the ultrathin layer and porous structure, the sheet-like GNS/PANi composites can deliver specific capacitances of 532.3 to 304.9 F/g at scan rates of 2 to 50 mV/s. And also, this active material showed very good stability with capacitance retention as high as ~99.6% at scan rate of 50 mV/s, indicating a great potential for using in supercapacitors. Furthermore, the effects of hydrothermal temperatures on the electrochemical performances were systematically studied and discussed.

Supercapacitors are considered as a promising energy storage device due to its high power density, long cycle life and low cost. However, the relative low energy density compared to rechargeable batteries excludes it from widely application as primary power sources. Considering its key techniques, searching for electrode materials with good electrochemical performances, are the main tasks for developing supercapacitors with high energy densities. Conducting polymers^1^,^2^,^3^, transition metal oxides^4^,^5^,^6^ and hydroxides^7^,^8^, which exhibit superior pseudocapacitive properties, are being extensively studied as electrode materials for supercapacitors with increased specific capacitance and high energy density. However, their rate capability, which rely on fast faradic redox reactions, are inhibited because these active materials are generally too insulating to satisfy the fast electron transport required by high rates^8^.

Graphene, which is monolayer of carbon atoms arranged in a honeycomb network, is becoming one of the most excellent matrixes because of its unique properties such as superior electronic conductivity, excellent mechanical flexibility, large surface area and high chemical stability^9^,^10^,^11^. In this regard, graphene is a good matrix for synthesis of functional nanomaterials to enhance their electroactivity and electronic conductivity^12^,^13^,^14^,^15^,^16^,^17^,^18^,^19^. Among these, the composites of graphene and conducting polymers are of scientific and industrial interests due to their enhanced properties arising from the high conductivity and synergistic π-π effect^18^. Generally, conducting polymers/graphene composites were fabricated by noncovalent combination methods via interaction between the π-bonds of the aromatic rings of conducting polymers and the graphitic structures of graphene. As a representative, polyaniline, an outstanding candidate for application as electrode materials of supercapacitor, is widely investigated in recent years due to its environmental stability, controllable conductivity by doping/de-doping process and easy synthesis, which is generally considered to promote the electrochemical capacitance of carbon materials^1^,^20^,^21^. For example, PANi coated carbon nanotubes and mesoporous carbon exhibit superior electrochemical performance^1^,^22^,^23^. And also, the composites of PANi and graphene are also widely prepared to improve the electrochemical properties by chemical or electrochemical polymerization and noncovalent functionalization^18^,^24^,^25^,^26^. However, most of these works are based on reduced graphene oxide (r-GO) as precursors. The electrochemical properties are much poorer compared with transitional metal oxides and hydroxides^6^,^27^,^28^. The reason is the serious aggregation problems of r-GO, which cannot disperse PANi homogeneously onto the surface of graphene with tight interfacial combinations. Thus, this will further restrict the electron transportation and electrolyte ions diffusion. The general strategies to overcome these problems are developing nanoporous or three-dimensional (3D) architectures composites^29^,^30^,^31^,^32^. For example, a free-standing 3D GNS/PANi composite film can show a specific capacitance as high as 740 F/g at 0.5 A/g^31^; and a 3D skeleton network of GNS wrapped PANi nanofibers can deliver a specific capacitance as high as 921 F/g^32^. Though these works reported very high specific capacitances, the 3D structures with macropores in micrometer size could not help to improve the volumetric energy density. Therefore, fabricating uniform and ultrathin layers of GNS/PANi composite with suitable porous structures as advanced electrochemical electrode materials remains a great challenge.

To overcome these, by controlling hydrothermal conditions, nanostructured PANi with different morphologies were composited with GNS and used as electrode materials of supercapacitors in this report. Specifically, ultrathin PANi layers with total thickness of 10–20 nm are uniformly composited with GNS by a two-step hydrothermal-assistant chemical oxidation polymerization process; while PANi nanofibers with diameter of 50~100 nm are obtained by a one-step direct hydrothermal process. The as synthesized sheet-like GNS/PANi composites show high specific capacitances up to 532.3 F/g at a scan rate of 2 mV/s, even at 50 mV/s, the specific capacitance can still as high as 304.9 F/g with good cyclic performances. This indicates that the method greatly improves the specific capacitance, rate capability and cycling stability.

## Results

### Morphology: hydrothermal process dependence

**Figure 1:**
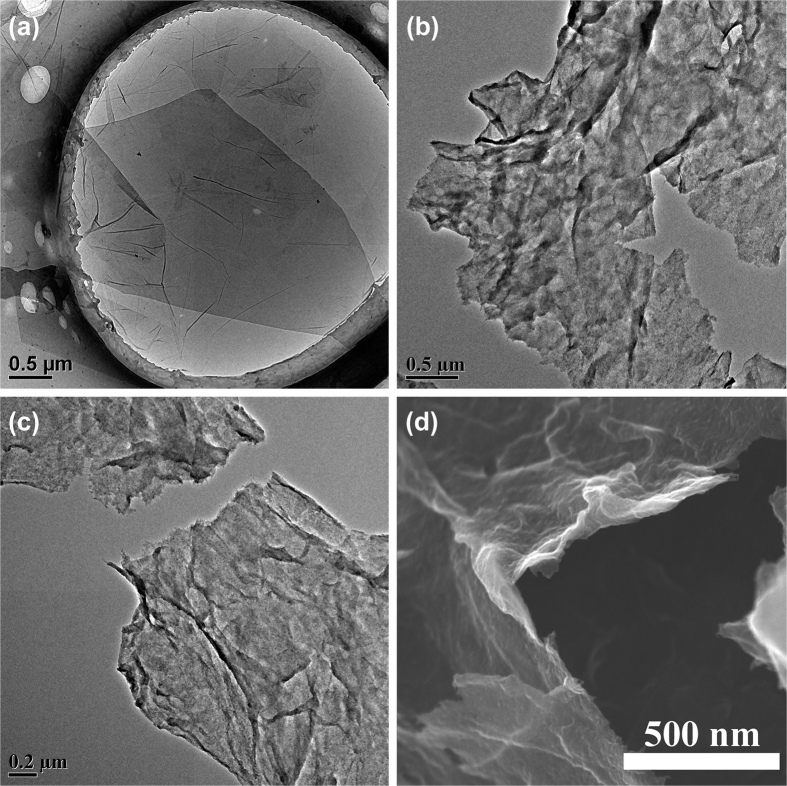
(**a**) TEM images of GO; (**b**) TEM image of GO/PANi composites; (**c**,**d**) TEM and SEM images of GNS/PANi composites prepared at 120 °C (S120).

The GNS/PANi composites were synthesized by hydrothermal-assistant chemical oxidation polymerization method. Figure 1 showed the TEM and SEM images of GO prepared by modified Hummer’s method and the sheet-like GO/PANi composites (precursors of sheet-like composites). It is observed that GO are mostly single to few layers with very large area and wrinkles on the surface (Fig. 1a). Figure 1b demonstrated that the uniform GO/PANi composites prepared by *in*-*situ* chemical oxidation polymerization method^33^,^34^. It is verified that PANi successfully coated onto GO. As shown in the literature, hydrothermal can be applied to prepare graphene with high efficiency. Here, we adopted the hydrothermal-assistant approach to synthesize GNS/PANi composites. The dark-green GO/PANi intermediate products were transferred to 100 mL of Teflon lined stainless steel autoclaves for hydrothermal reaction at different temperatures^35^,^36^,^37^, during which GO was converted to GNS as shown in Fig. S1^35^,^38^,^39^. The content of GNS in the final products is about 20 wt. % based on the final weight of the composites. As shown in Fig. 1c, the obtained samples prepared at 120 °C (named as S120) illustrated a sheet-like microstructure with rough surface, similar to that of GO/PANi composites. The SEM image exhibited that the thickness of the GNS/PANi composites is about 10–20 nm with very rough surface morphology (Fig. 1d), consistent with the SEM images. The ultrathin layered structures can greatly shorten the distance of ions transportation and increase the ratio of composites to attend the electrochemical reactions^40^. Additionally, the rough surface can also improve the wettability of the electrochemical interface and increase the electrochemical active sites when used as electrode materials for supercapacitors^40^,^41^. Further increasing the hydrothermal temperature to 150 and 180 °C (named as S150 and S180), the microstructures of products are similar to that of composites synthesized at 120 °C. However, in the controlled experiment when GO was firstly reduced to GNS by the hydrothermal process, the GNS/PANi composites exhibited serious aggregations (Fig. S2), which will not be beneficial for the electrochemical performance.

**Figure 2:**
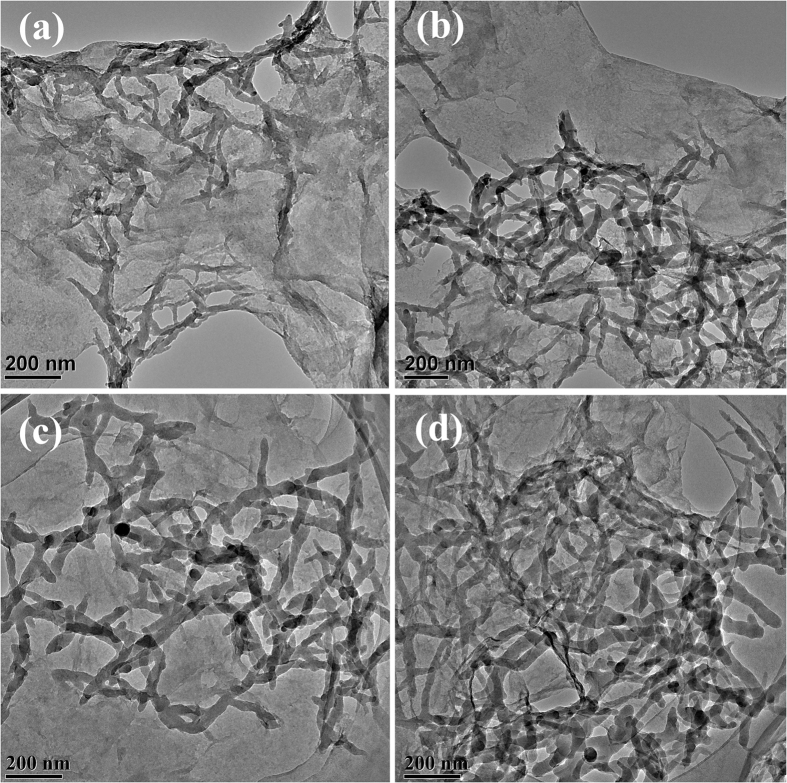
TEM images of sample A120 (**a**,**b**), A150 (**c**) and A180 (**d**) by direct hydrothermal treatment.

By controlling the hydrothermal conditions, if we use direct hydrothermal process, instead of hydrothermal-assistant chemical oxidation polymerization, GNS/PANi nanofiber composites will be obtained. As shown in Figs 2 and S3, PANi nanofibers with diameter of 50~100 nm and length of several micrometers are directly intercalated into GNS. Similar microstructures are observed by different hydrothermal temperature treatment. SEM images of the GNS/PANi nanofibers exhibit that PANi nanofibers are tightly and uniformly connected with GNS (Fig. S3). And also, many pores are observed in the SEM images. This microstructure may also greatly improve the electrochemical properties due to facile electrolyte diffusion and increase the ratio of active materials to attend the electrochemical reactions.

### Structural Characterization and Properties

**Figure 3:**
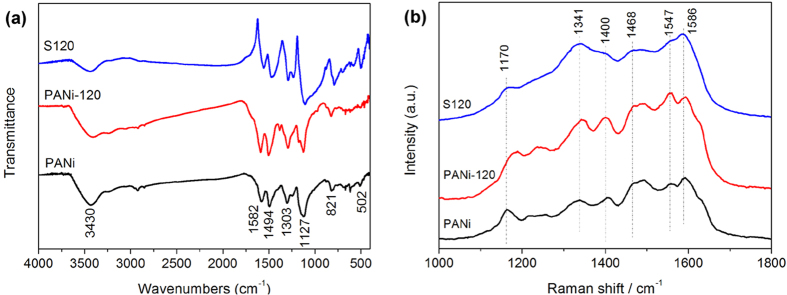
(**a**) FT-IR and (**b**) Raman spectra of sample S120, polyaniline and graphene/polyaniline composites prepared by chemical polymerization.

In order to evaluate the structural stability of PANi treated or synthesized by hydrothermal process, we further characterized Fourier transformation infrared (FT-IR) and Raman spectrum of the GNS/PANi composites. As a control, PANi and GNS/PANi (PANi-120) synthesized by chemical polymerization has used as standard sample for comparison. As shown in Fig. 3a, the peak positions of all three samples are the same, indicating that the hydrothermal treatment did not affect the FTIR results, which indirectly proved that the PANi is stable at this moderate temperature. Further increasing the hydrothermal temperature to 150 and 180 °C, the FTIR curves are also similar with the chemical polymerization one. The characteristic peaks at 1582 and 1494 cm^−1^ are due to the stretching vibration of quinoid ring and benzenoid ring, respectively. The bands at 1303 and 1250 cm^−1^ can be assigned to C-H stretching vibration with aromatic conjugation. It is generally accepted that the absorption peak near 1127 cm^−1^ results from the N = Q = N (Q denotes quinoid ring) stretching mode and is an indication of electron delocalization in PANi; which is also a character of the delocalization of electrons in the PANi backbones, indicating the success of the polymerization of aniline. The peak centered at 821 cm^−1^ is attributed to the vibration of C-C and C-H in the benzenoid structures. Figure S5 also showed the FTIR curves of GNS/PANi nanofiber composites. It can also approve that all peaks can be assigned to the characteristic peaks of PANi, but slightly shifts are observed compared to the pure PANi by chemical polymerization. This may be caused by the structure changes of PANi in the direct hydrothermal process. The Raman spectra show no significant structural changes occurring during the hydrothermal treatment (Fig. 3b), except for the relative intensity of the peaks. The spectrum of neat PANi show bands at 1170, 1341, 1547 and 1620 cm^−1^ corresponding to C-H bending of the quinoid ring, C-N stretching of the bipolaron structure, N-H bending of the bipolaronic structure and C-C stretching of the benzenoid ring, respectively. Because the quinoid rings in the PANi have a similar atomic structure with the C6 rings in GNS, this will allow for a strong π-π stacking interaction and benefit for electronic transmission between them^41^,^42^.

## Discussion

**Figure 4:**
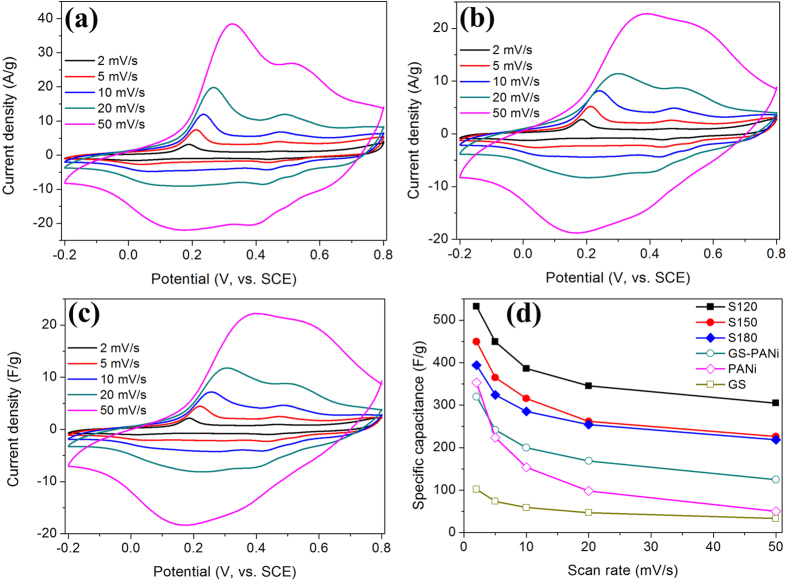
CV curves of graphene/polyaniline composites: (**a**) S120; (**b**) S150; (**c**) S180; (**d**) The variation of the specific capacitance of GNS/PANi composites at different scan rates.

The ultrathin sheet-like GNS/PANi composites were re-dispersed into 10 mL absolute ethanol for preparing the working electrodes (all electrochemical performances of GNS/PANi nanofiber composites could be found in Figs S8–S10, see Supplementary Information Section). The electrochemical measurements were carried out in a three-electrode cell system with a platinum wire as counter electrode and saturated calomel electrode as reference electrode. The electrolyte is 1 M H_2_SO_4_ solution. The electrochemical performance of the electrode materials was analyzed using cyclic voltammetry (CV) methods. Figure 4a–c show the CV curves of sample S120, S150 and S180 at scan rates of 2, 5, 10, 20 and 50 mV/s in the potential range from −0.2 to 0.8 V. All of the composite electrodes show two pair of redox peaks (~0.4/0.5 V and ~0.1/0.2 V) at low scan rates (2–10 mV/s), which can be ascribed to a comprehensive effect of the changing in PANi structures and the remained oxygenated groups of the GNS-based nanosheets^43^. When increasing the scan rate to 20 mV/s, the CV curves are still similar with the low rates ones. The specific capacitances of the electrodes can be calculated from CV curves^24^,^44^. The specific capacitances are about 532.3, 449.2 and 393.8 F/g for S120, S150 and S180 at 2 mV/s. Further enhancing the scan rate to 50 mV/s, the GNS/PANi composites still deliver capacities of 304.9, 226.2 and 218.5 F/g. The sample that treated at lowest temperature (S120) has the best electrochemical performance compared with that at higher temperatures (S150 and S180), though higher temperature can lead to a better reduction degree^35^. This can be attributed to that the PANi may lose electroactivity under high heat treatment^45^,^46^, which can be proved by the electronic conductivity of the GNS/PANi composites treated at different hydrothermal procedures (the electronic conductivities are 3.4, 0.11 and 5.6 × 10^−4^ S/m for S120, S150 and S180, respectively).

**Figure 5:**
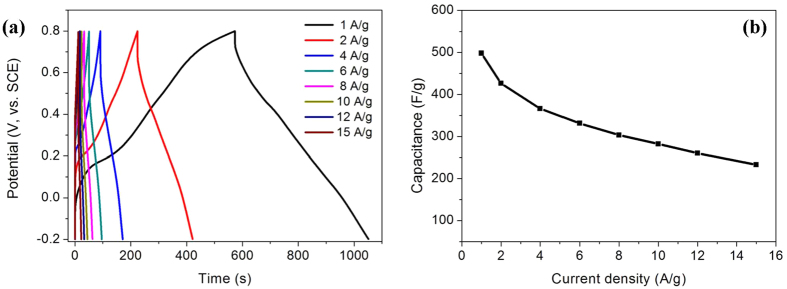
The galvanostatic charge-discharge curves of S120 at different current densities and its rate performances.

We also compared the performances of the samples with that of GNS/PANi prepared by chemical polymerization at room temperature, pure PANi and GS (Fig. 4d). These samples showed electrochemical capacitances of 320, 353 and 101.9 F/g at a scan rate of 2 mV/s, which are much lower than that of GNS/PANi obtained by hydrothermal process. To quantify the capacitances of the best sample (S120), the galvanostatic charge-discharge measurements were further made. In Fig. 5a, the charge-discharge curves show well triangular shapes at various current densities (from 1 to 15 A/g), demonstrating good capacitive property and reversibility. The capacitances obtained from here are plotted against the current densities in Fig. 5b. S120 give capacitances of 497.9 to 232.8 F/g from 1 to 15 A/g, showing that the specific capacitances and rate capability are good compared with former work due to GNS modifications and the sheet-like structures^20^,^24^,^25^,^36^.

**Figure 6:**
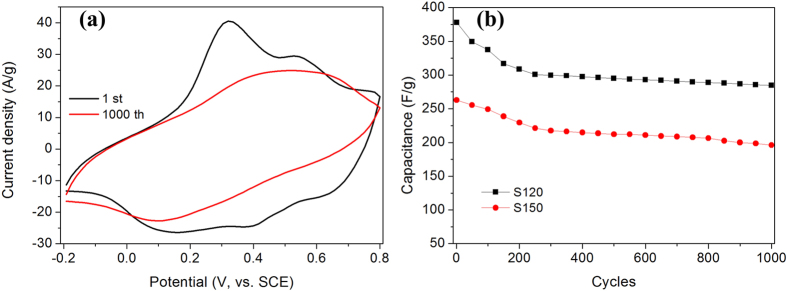
(**a**) CV curves of sample S120 at 1st and 1000th cycles at a scan rate of 50 mV/s; (**b**) Cycle life of S120 and S150 at 50 mV/s.

**Figure 7:**
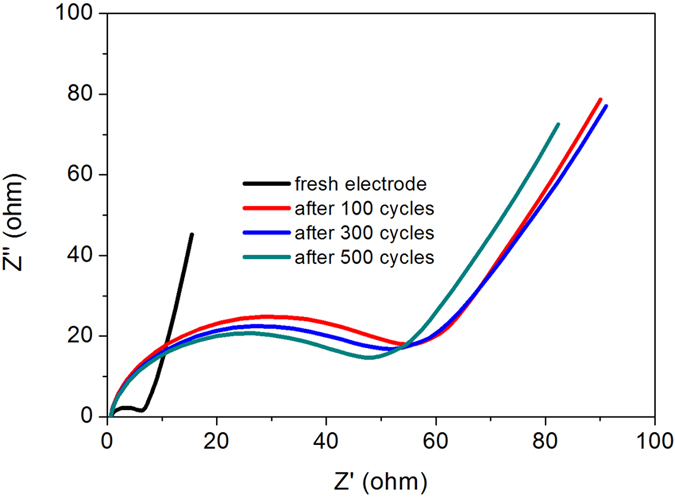
Nyquist plots of fresh electrode and after hundredth cycles for S120.

We further cycled sample S120 and S150 at scan rate of 50 mV/s (Fig. 6). Figure 6a demonstrated the 1st and 1000th CV cyclic curves of S120. Apparently from the area of the curves, there is a larger capacitance loss during these cycles. And also, the redox peaks disappeared indicating that the PANi occur some changes in some extent. The sample S120 retained a capacitance of 299.4 F/g after 300 cycles; after, the capacitance retention is as high as ~99.6% and the capacitance retained 284.6 F/g after 1000 cycles. In order to further understand the capacitance loss, Nyquist plots of fresh electrode and electrode after hundredth cycles of S120 were characterized. As shown in Fig. 7, the estimates of equivalent series resistance (ESR) is ~0.65 and charge transfer resistance (*R*_ct_) is ~6.8 Ω, indicating good conductivity and low internal resistance. While, after 100, 300 and 500 cycles, *R*_ct_ increased to 56.6, 52.3 and 48.5 Ω, which can explain the initial capacitance loss and high capacitance retention after 300 cycles.

## Conclusion

In summary, we have synthesized nanostructured PANi composited with GNS in different morphologies as electrode materials for supercapacitors. As a result, ultrathin PANi layers with total thickness of 10–20 nm and PANi nanofibers with diameter of 50–100 nm are uniformly composited with GNS by controlling hydrothermal conditions. The as synthesized sheet-like GNS/PANi composites show high specific capacitances up to 532.3 F/g at a scan rate of 2 mV/s, even at 50 mV/s, the specific capacitance can still as high as 304.9 F/g with good cyclic performances (capacitance retention is as high as ~99.6%). The reason is that the ultrathin layer-structure is of great benefit to active material participating in the interfacial electrochemical reactions.

## Materials and Methods

Graphite was purchased from Alfa Aesar (−325 mesh). All chemicals were of analytic grade and used without further purification. GO was prepared from graphite flakes by a modified Hummers method. 1.0 g of graphite flakes, 1.0 g of NaNO_3_ and 46 mL of concentrated H_2_SO_4_ were mixed together in an ice bath for 4 h. Then 6.0 g of KMnO_4_ was added slowly into the solution. Afterwards, the ice bath was removed and the suspension was stirred for another 4 days. After adding 100 mL of distilled water dropwise, the suspension was heated in oil bath at 98 °C for 30 min. Then the suspension was further treated with 200 mL of warm water (~60 °C) and 10 mL of H_2_O_2_ (30%). The mixture was centrifuged at 4000 rpm and washed with diluted HCl and water to neutral. Finally, a homogeneous GO aqueous dispersion (0.5 mg/mL) was obtained for further using.

The GNS/PANi nanosheet composites were synthesized by a simple hydrothermal-assistant chemical polymerization. Firstly, 100 μL of aniline and 0.5 mL of concentrated HCl were added into 60 mL of the above GO suspension (0.5 mg/mL) followed by stirring for more than 30 min. Afterward, 5 mL of ammonium persulfate (APS, 1 mmol) aqueous solution was added into the above reaction system and kept at 0–4 °C for 4–6 h. Then, the obtained dark-green suspension was transferred to 100 mL of Teflon lined stainless steel autoclaves for hydrothermal reaction at 120, 150 and 180 °C for 6 h. Finally, the obtained products were washed with distilled water and absolute ethanol for several times and re-dispersed into 10 mL absolute ethanol for further use. The materials were denoted as S-temperature, such as S120 indicating the sample was synthesized at hydrothermal temperature of 120 °C. For GNS/PANi nanofiber composites, the raw materials were transferred to Teflon lined stainless steel autoclaves for one-pot hydrothermal chemical polymerization directly. Finally, the nanofiber composites were denoted as A-temperature, such as A120 indicating the sample synthesized as hydrothermal temperature of 120 °C. The pure polyaniline was also prepared by chemical oxidation polymerization process.

### Materials Characterizations

Transmission electron microscopy (TEM) was performed on JEM-2100F Electron Microscope with an accelerating voltage of 200 kV. Field-emission scanning electron microscope (FE-SEM) was performed on JSM-6700F at an acceleration voltage of 10.0 kV. FT-IR and Raman spectroscopy were recorded on a Nicolet iZ10 and DXR Raman Microscope with 532 nm excitation length, Thermal Scientific Co., USA, respectively.

### Electrochemical Measurements

In electrochemical test, the active electrode was assembled into a three-electrode cell system. The work-electrode was prepared according to the following steps. The re-dispersed products were dropped onto 1 cm × 1.5 cm stainless steel mesh, and then dried under vacuum at 60 °C for 12 h and pressed at 10 MPa. The mass loading of active materials is in the range of 1.5~2.0 mg. The electrochemical behavior was characterized within a potential window of −0.2 to 0.8 V vs. SCE reference electrode. Platinum wire was used as a counter electrode. All the electrochemical experiments were carried out using CHI 760E electrochemical stations.

### Capacitances Calculation

The specific capacitances based on CV tests were calculated by the following equation:


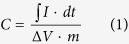


where *I* is the applied current, Δ*V* indicates the potential window for one sweep segment, d*t* is the differential time, and *m* is mass loading of the active material.

Also, the specific capacitances could be collected from galvanostatic charge-discharge curves by the following equation:


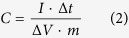


where I, Δ*V*, Δ*t*, and *m* are the discharge current, voltage range, discharge time, and active material mass, respectively.

## Additional Information

**How to cite this article:** Wang, R. *et al*. Hydrothermal synthesis of nanostructured graphene/polyaniline composites as high-capacitance electrode materials for supercapacitors. *Sci. Rep.* **7**, 44562; doi: 10.1038/srep44562 (2017).

**Publisher's note:** Springer Nature remains neutral with regard to jurisdictional claims in published maps and institutional affiliations.

## Supplementary Material

Supplementary Information
