# Supplementary material for: Hydrothermal synthesis of nanostructured graphene/polyaniline composites as high-capacitance electrode materials for supercapacitors
Source: Sci Rep. 2017 Mar 14;7:44562. doi: 10.1038/srep44562 (PMC5349513; doi:10.1038/srep44562)
Supplement: Supplementary Information [file srep44562-s1.doc]

# Hydrothermal synthesis of nanostructured graphene/polyaniline composites as high-capacitance electrode materials for supercapacitors

Ronghua Wang^1*^, Meng Han^2^, Qiannan Zhao^2^, Zonglin Ren^2^, Xiaolong Guo^2^, Chaohe Xu^2^*, Ning Hu^2^, & Li Lu^3^

E-mail: [wangrh@cqu.edu.cn](mailto:wangrh@cqu.edu.cn); [xche@cqu.edu.cn](mailto:xche@cqu.edu.cn);

Affiliation:

^1^ College of Materials Science and Engineering, Chongqing University, No. 174 Shazhengjie Road, Chongqing 400044, P.R. China

^2^ College of Aerospace Engineering, and The State Key Laboratory of Mechanical Transmissions, Chongqing University, No. 174 Shazhengjie Road, Chongqing 400044, P.R. China

^3^ Department of Mechanical Engineering, National University of Singapore, 2 Engineering Drive 3, Singapore 117581, Singapore

**Supplementary Figures**


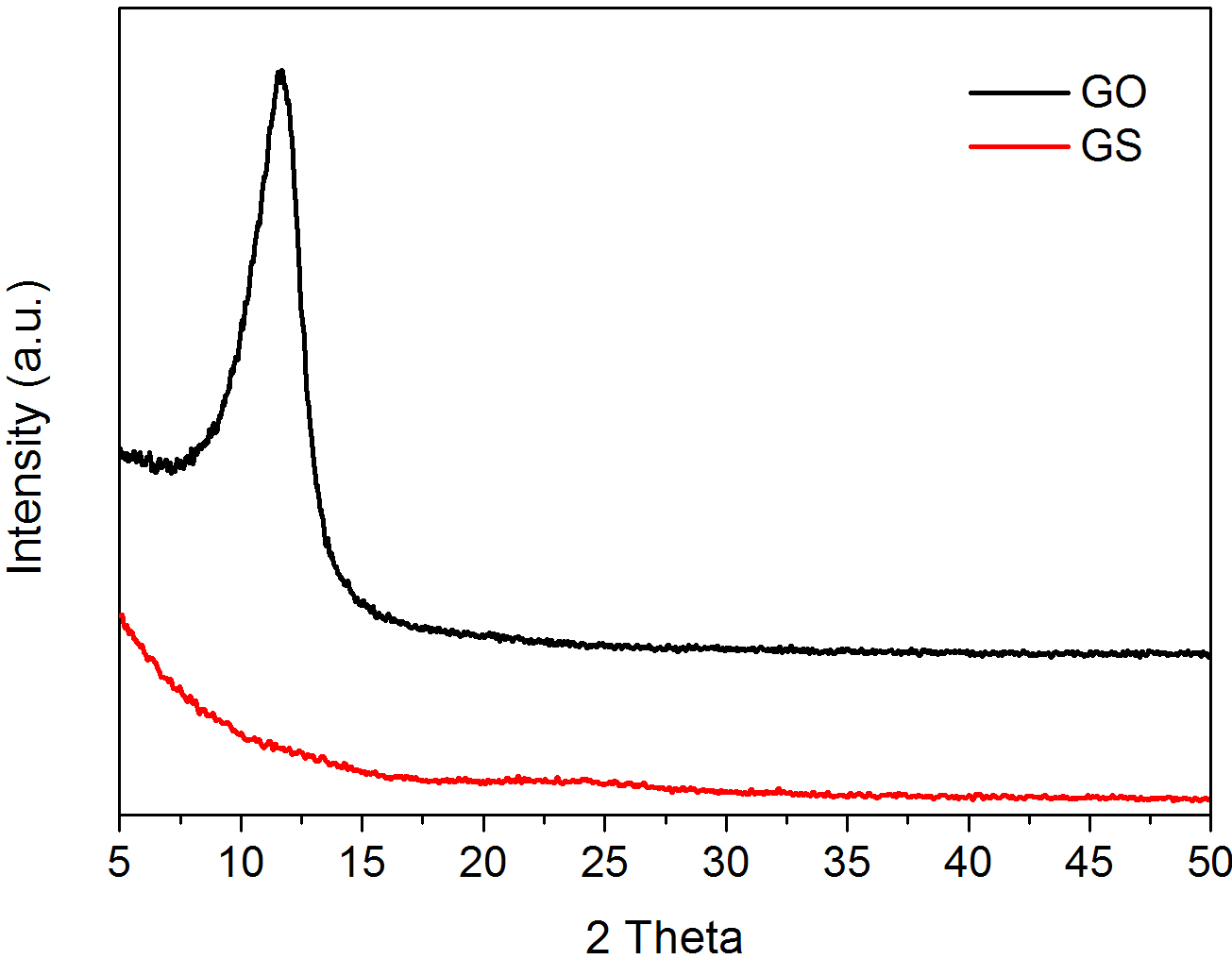


**Figure S1.** XRD curves of GO and GS obtained by hydrothermal process.

**
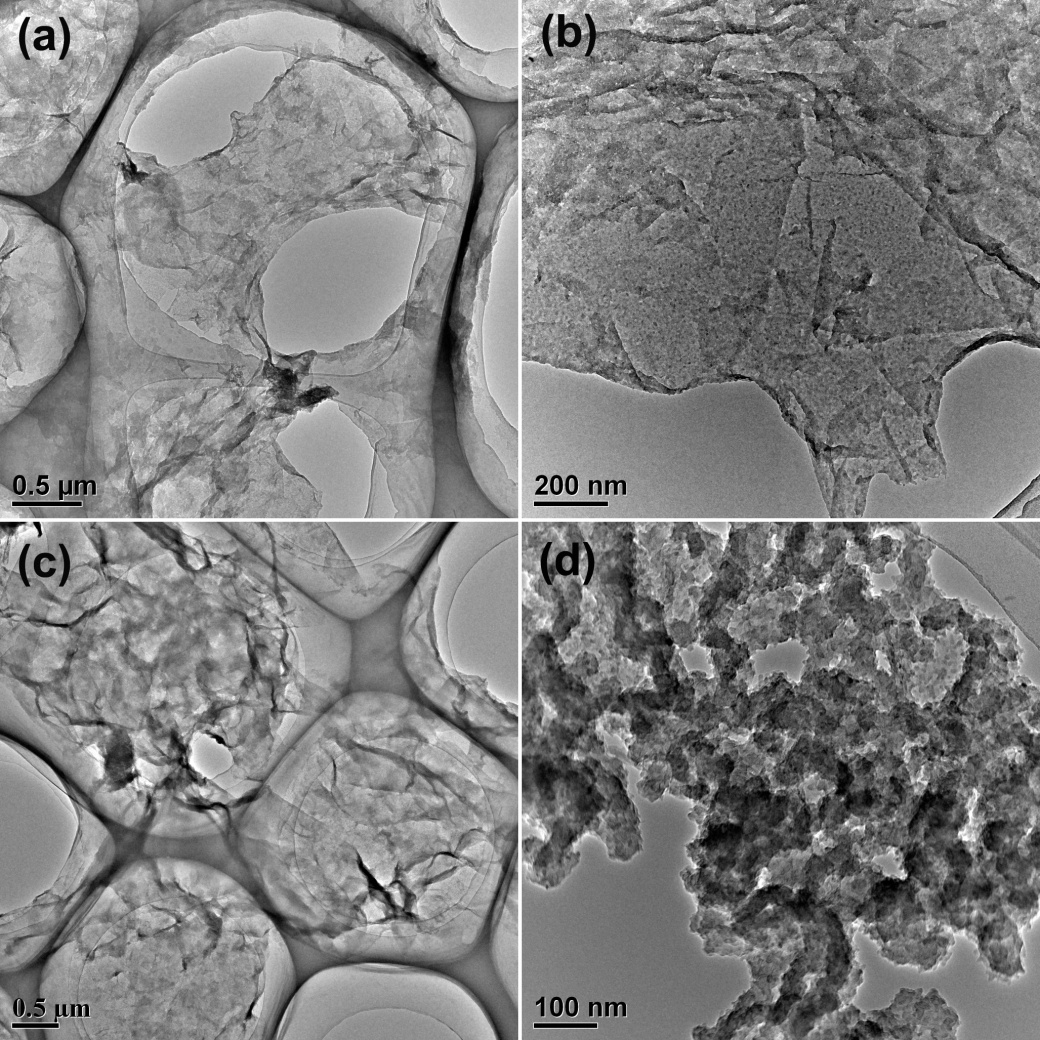
**

**Figure S2.** (a-c) TEM images of S120, S150 and S180, respectively; (d) TEM image of GS/PANi composites prepared by chemical polymerization.


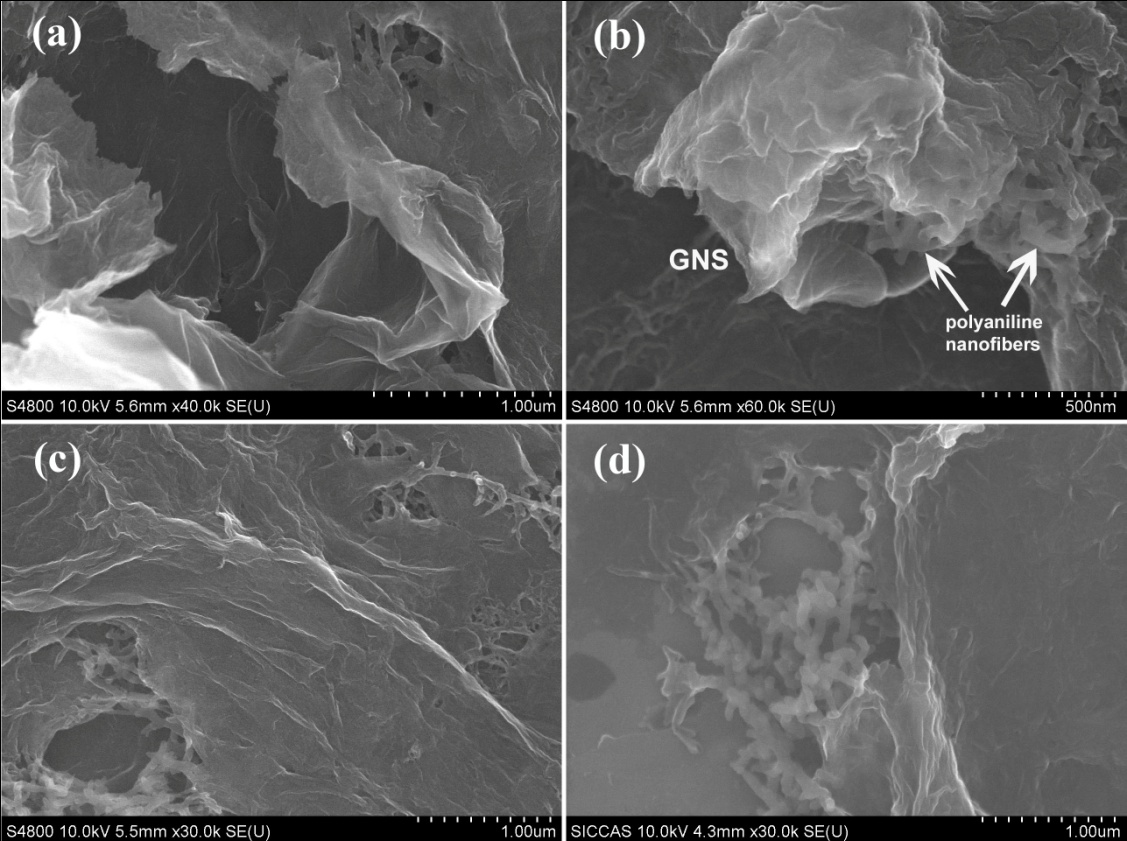


**Figure S3.** SEM images of sample A120 (a-b)，A150 (c) and A180 (d), respectively.


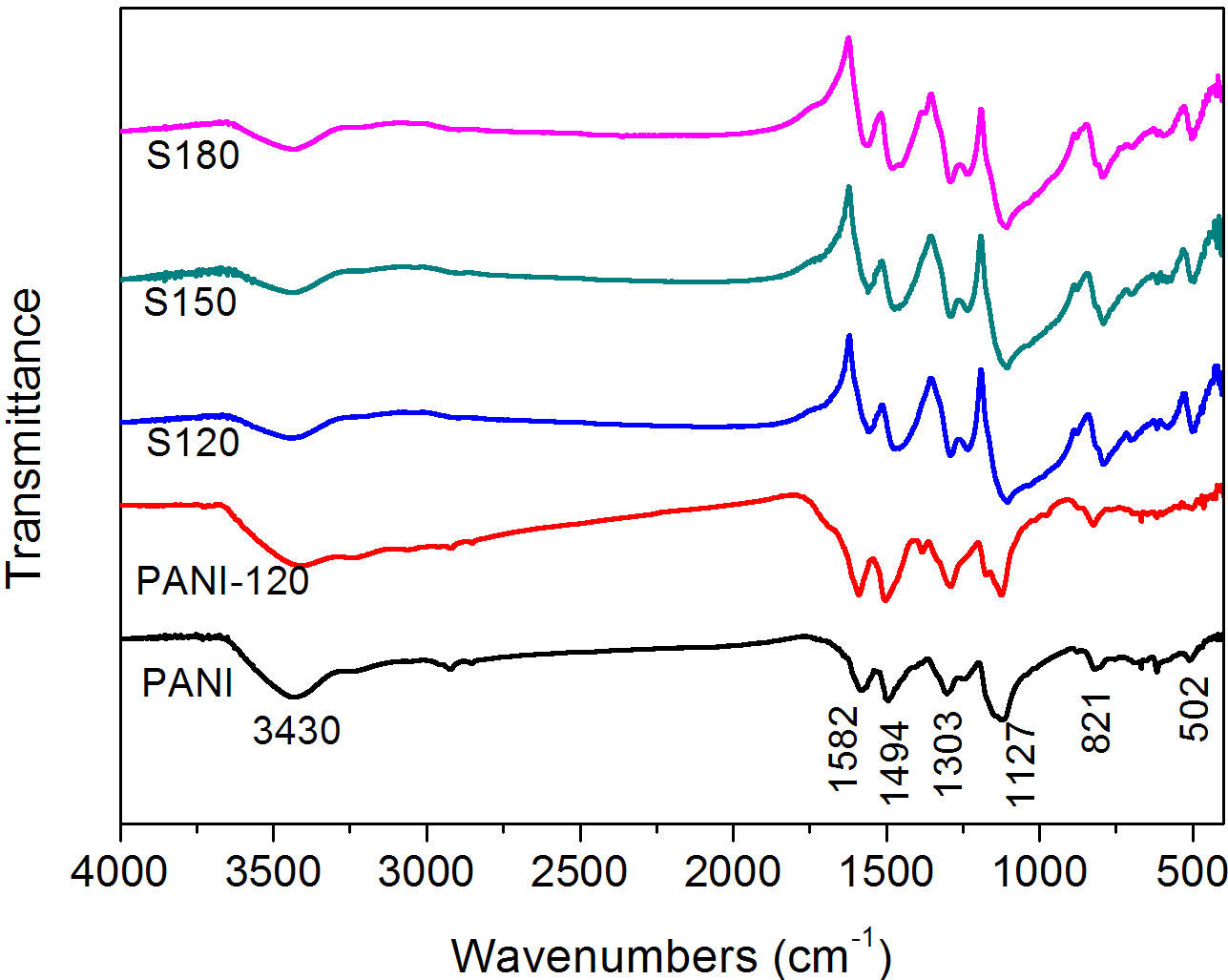


**Figure S4.** FT-IR spectra of the graphene/polyaniline nanosheet composites.


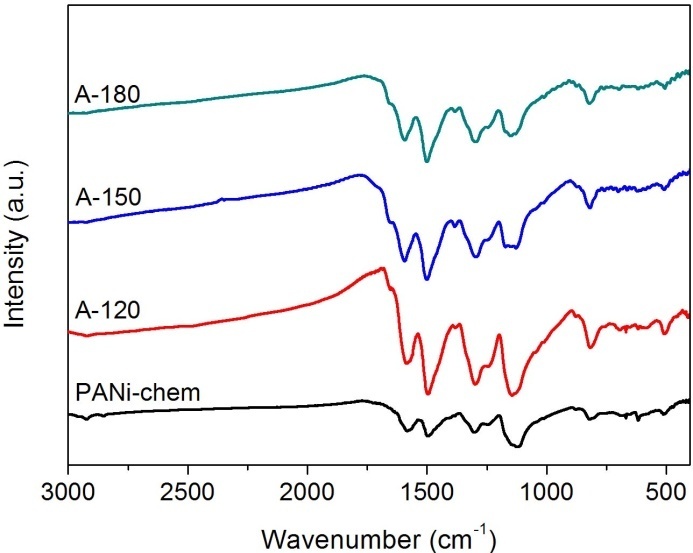


**Figure S5**. FT-IR spectra of the graphene/polyaniline nanofiber composites.


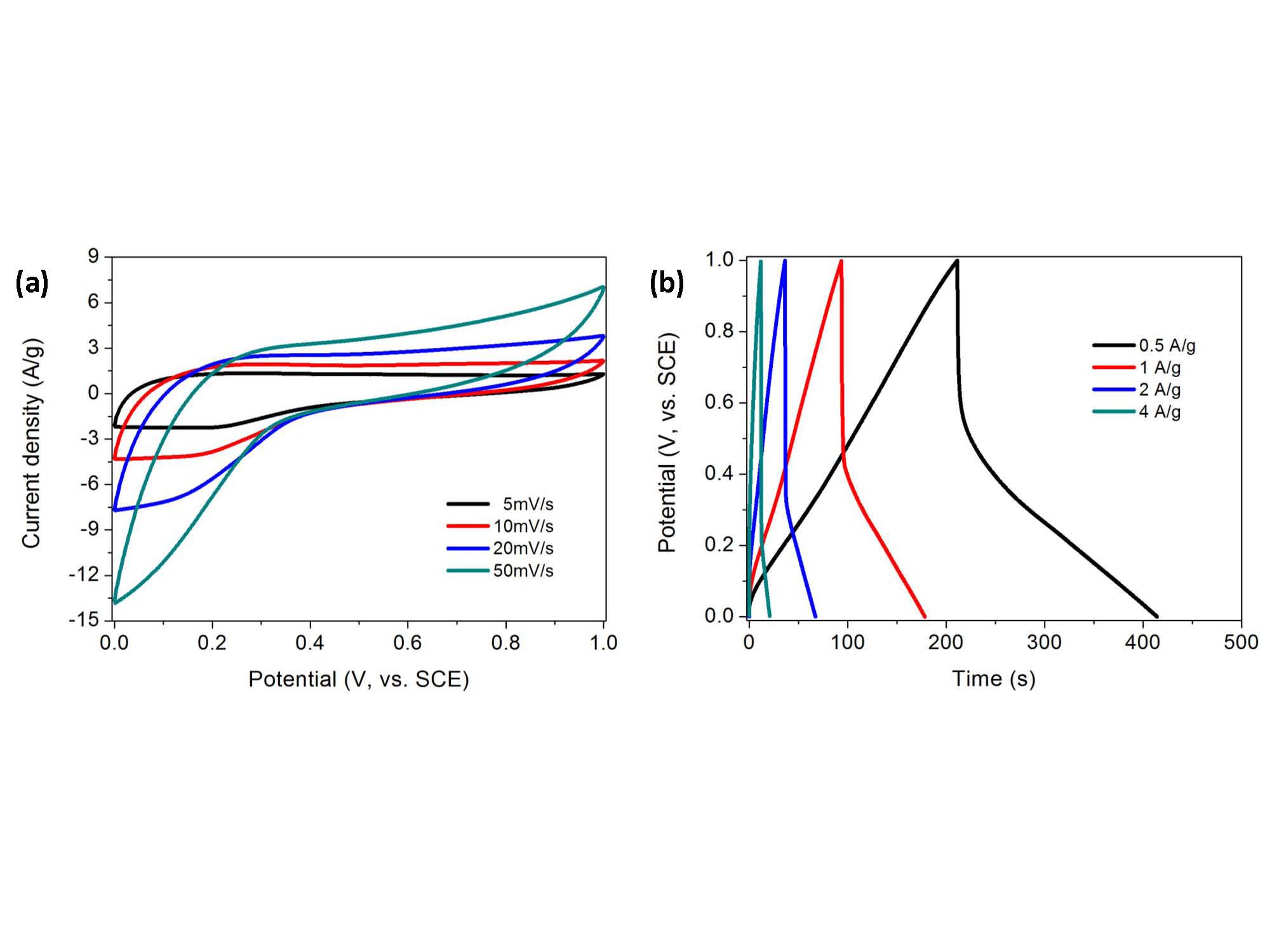


**Figure S6.** CV curves and galvanostatic charge-discharge curves of sheet-like GS/PANi // GS/PANi symmetric supercapacitor. The calculated specific capacitances based on the total mass of active materials in both electrodes are 106.9, 93.5, 74.5 and 49.8 F/g at current densities of 0.5, 1, 2 and 4 A/g, respectively. The formula is in following:

$C=\frac{I\cdot\Delta t}{\Delta V\cdot M}$ (S1)

where I, Δ*V,* Δ*t,* and *M* are the discharge current, voltage range, discharge time, and mass loading of active materials in anode and cathode electrodes, respectively.


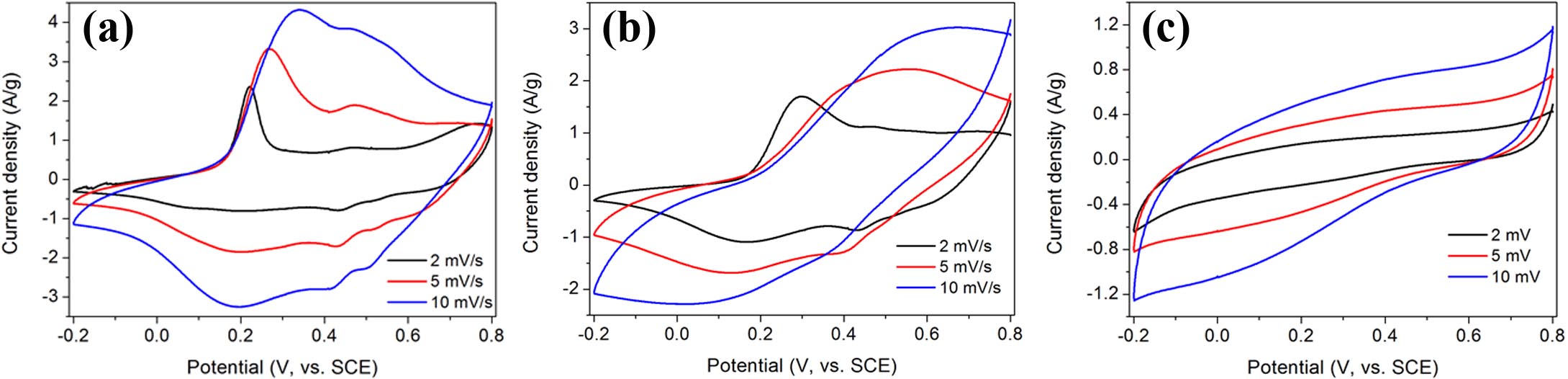


**Figure S7.** (a)CV curves of graphene/polyaniline composites prepared by chemical polymerization; (b) CV curves of polyaniline.


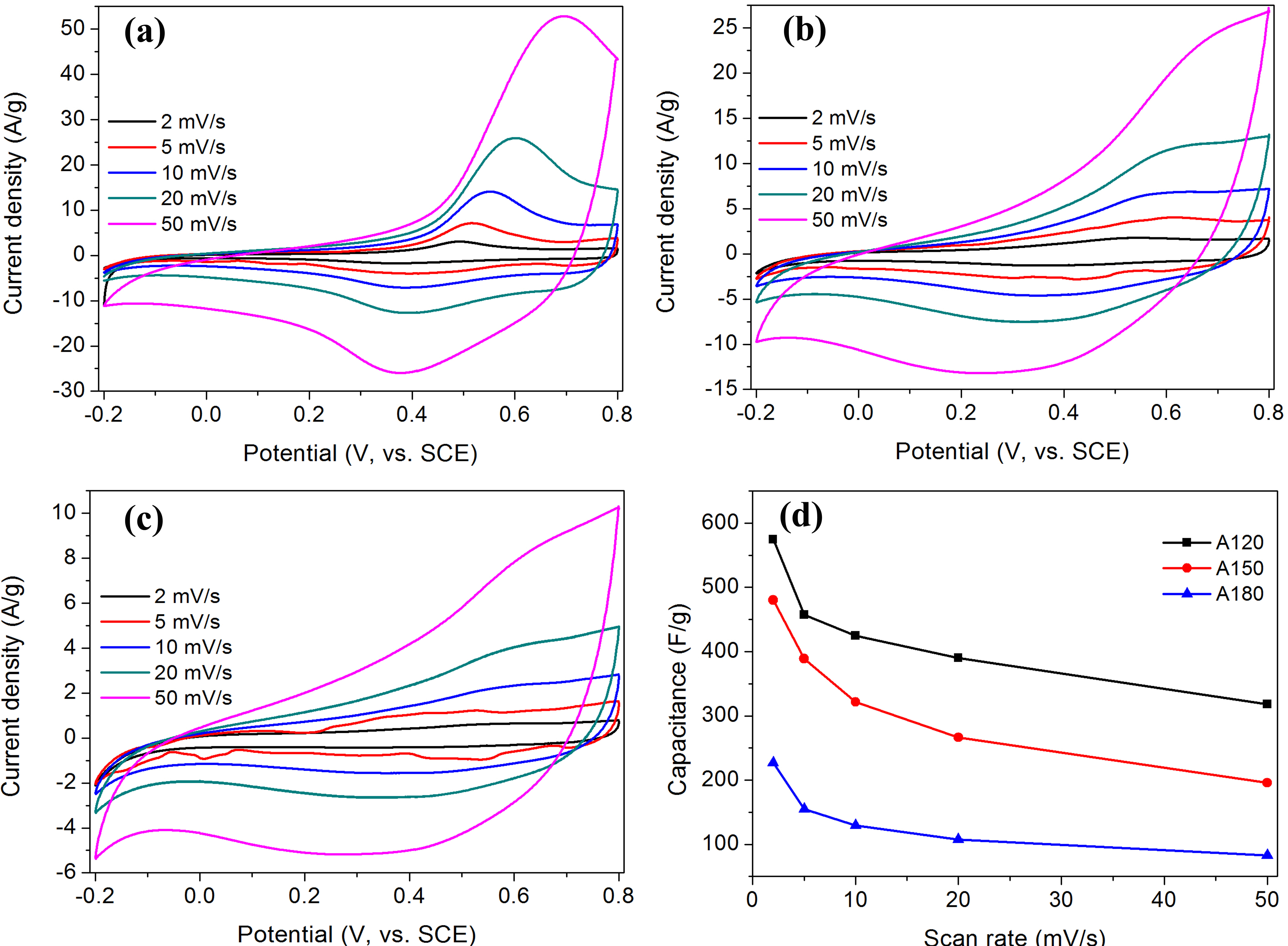


**Figure S8**. CV curves of graphene/polyaniline nanofiber composites：(a)A120；(b)A150；(c)A180；(d) The variation of the specific capacitance of graphene/polyaniline nanofiber composites at different scan rates. The specific capacitances of A120 (the best one of GNS/PANi nanofiber composite) were as high as 574.6, 457, 425, 389.9 and 317.8 F/g at scan rate of 2, 5, 10, 20 and 50 mV/s.

**
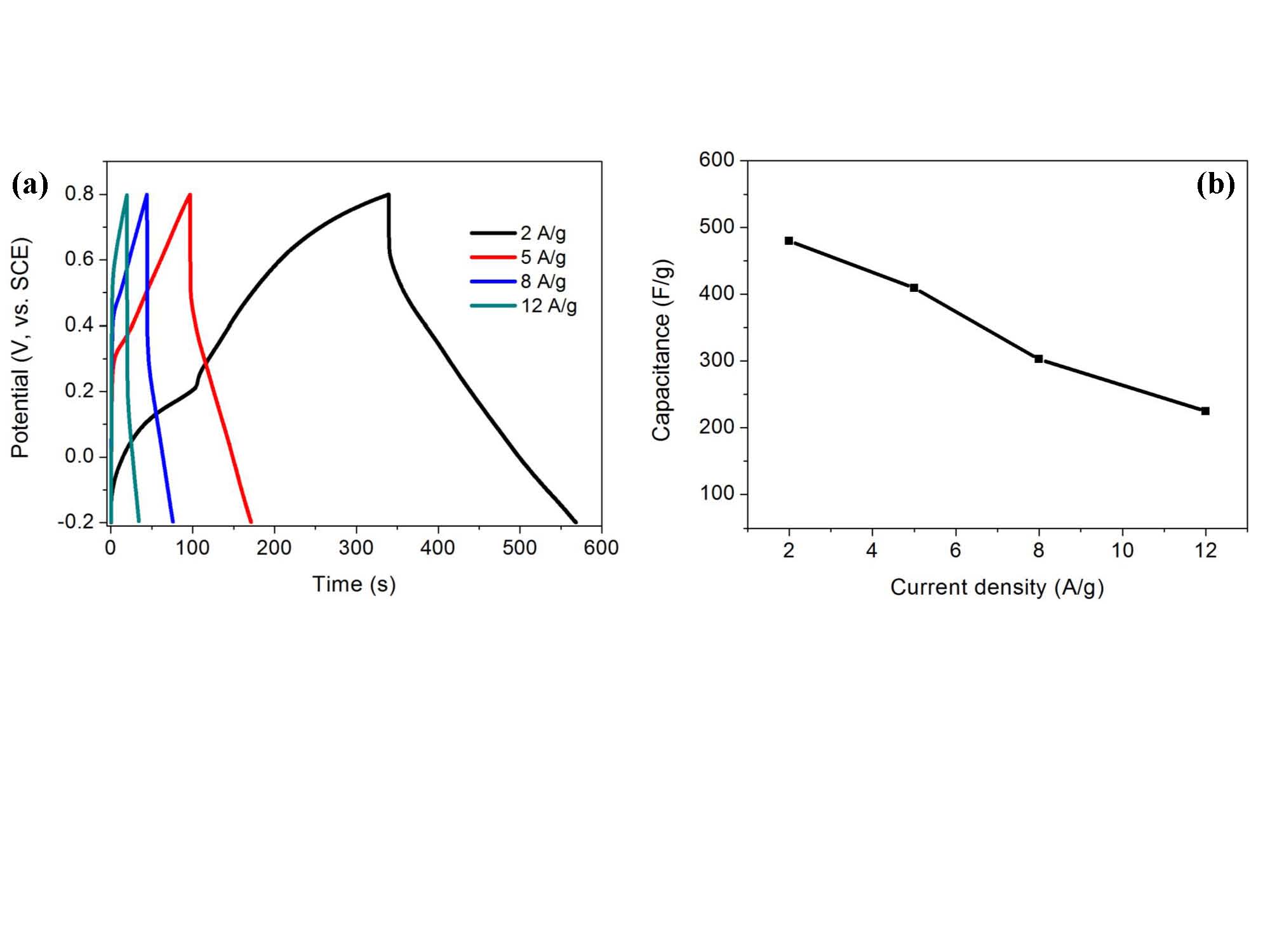
**

**Figure S9**. The galvanostatic charge-discharge curves at different current densities and its rate performances of sample A120. The specific capacitances were 479.9 to 224.5 F/g at current densities of 2 to 12 A/g.


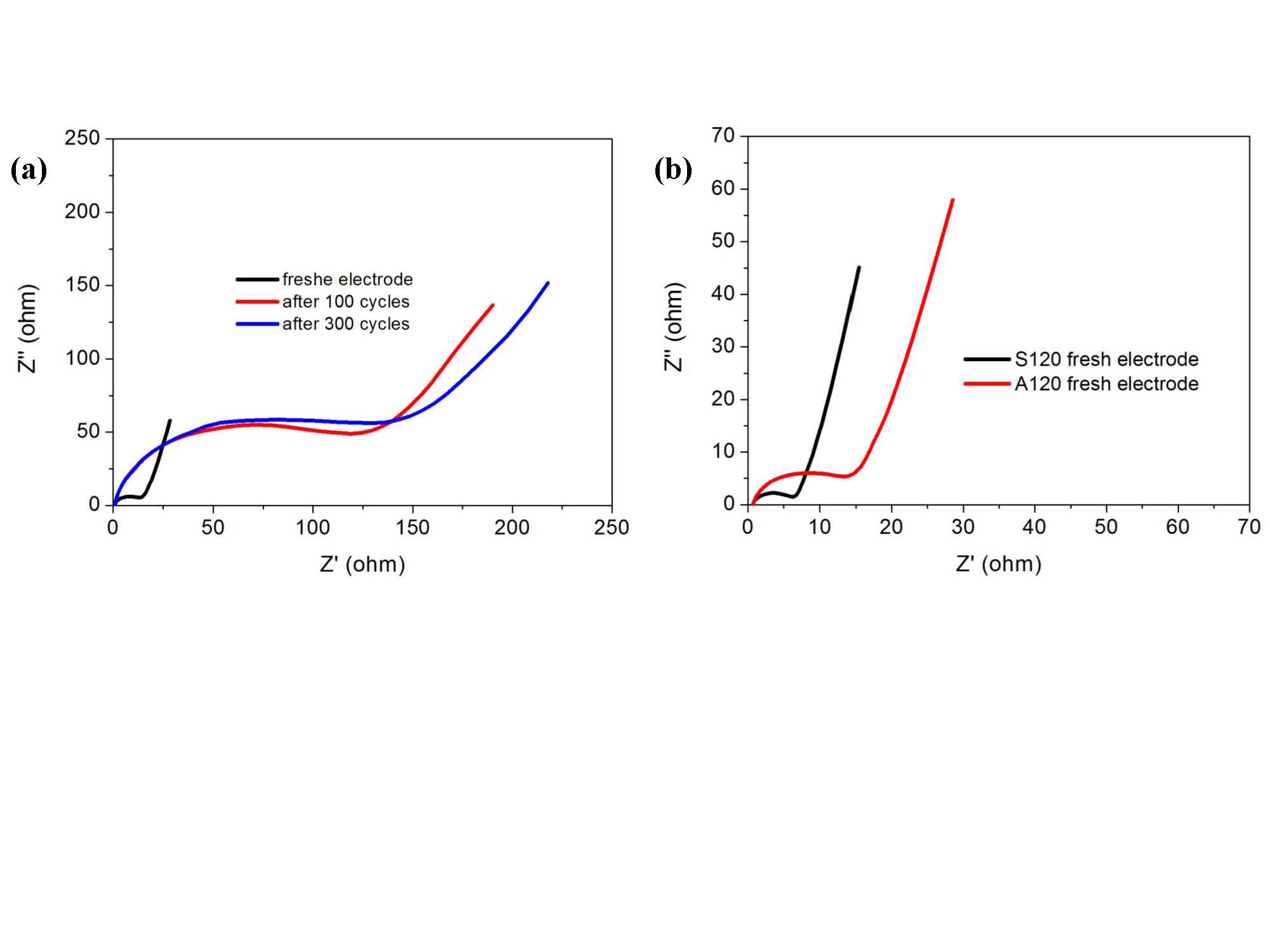


**Figure S10.** (a) Nyquist plots of fresh electrode and after hundredth cycles for sample A120; (b) Comparison Nyquist plots of sample S120 and A120. The estimate ESR is ~0.79 Ω and R_ct_ is ~12.9 Ω; and after 100 and 300 cycles, R_ct_ was increased to 124.5 and 143.5 Ω, respectively. While, the R_ct_ of A120 is larger than that of S120, indicating that the GNS/PANi nanofiber composites synthesized by direct hydrothermal process give a relative low intrinsic electronic conductive of PANi.
